# Prognostic criteria for the management of temporomandibular disorders using arthrocentesis with normal saline and arthrocentesis with normal saline and platelet-rich plasma

**DOI:** 10.25122/jml-2021-0240

**Published:** 2022-05

**Authors:** Ahmad Syed Ansar, Khan Munna, Asif Iqbal, Faisal Mohammad, Alam Naved, Hasan Shamimul

**Affiliations:** 1. Department of Oral and Maxillofacial Surgery, Jamia Millia Islamia Central University, New Delhi, India; 2. Department of Electrical Engineering, Faculty of Engineering and Technology, Jamia Millia Islamia Central University, New Delhi, India; 3. Department of Electrical Engineering, Vivekananda Global University, Rajasthan, India; 4. Department of Computer Sciences, Jamia Hamdard, New Delhi, India; 5. Department of Oral Medicine and Radiology, Faculty of Dentistry, Jamia Millia Islamia Central University, New Delhi, India

**Keywords:** arthrocentesis, bite force, platelet-rich plasma, temporomandibular joint disorders, TMDs – temporomandibular disorders, PRP – platelet-rich plasma, RDC – research diagnostic criteria

## Abstract

Temporomandibular joint disorders (TMDs) are ailments affecting the jaws and allied structures, resulting in many pathologies (TMJ hypermobility, internal disc derangement, bone changes, degenerative disorders, and ankylosis). Pain, clicking or crepitus, restricted range of motion, deranged jaw function, and deflected or deviated mouth opening and closing are the commonly observed manifestations in TMDs. Internal derangement refers to an aberrant relation of the articular disc to the condyle and fossa, respectively. Conventional therapies highlight the role of non-invasive conservative treatment strategies, namely joint unloading, anti-inflammatory drugs, and physiotherapy. Current literature has emphasized the use of corticosteroids and platelet-rich plasma (PRP) as treatment strategies in TMDs. This study aimed to evaluate whether intra-articular injection of PRP after normal saline lavage in TMJ minimizes the symptoms of TMDs, as compared to injection of normal saline. Thirty patients with TMD according to research diagnostic criteria (RDC) were selected. One group received arthrocentesis with normal saline, and the other group received arthrocentesis with PRP injection. The patients were assessed for pain, maximum inter-incisal mouth opening, bite force, and TMJ sounds. TMDs treated by PRP injection had slightly better results. More studies are required to substantiate the outcome. Injections of PRP were more effective in reducing the symptoms than arthrocentesis with normal saline.

## INTRODUCTION

Chronic facial pain is a common cause of disability in patients' daily activities. Management of pain requires medical and surgical procedures by health professionals. This raises the need for multidisciplinary treatment due to the complex character of complaints. Temporomandibular joint pain occurs in the movement of the mandible when chewing and speaking, increasing the pain even further [1–4].

Temporomandibular disorders (TMD) can be divided into muscle and articular categories. However, differences between the two are sometimes difficult to differentiate because muscle disorders may often camouflage articular disorders, or they may stay together. Myogenic complications include myalgia (myofascial pain, fibromyalgia), myospasm, isolation, and fibrosis/contracture. Articular problems include synovitis/capsulitis, inflammation of the joints, trauma/fractures, arthritis, and neoplasm [5].

Modification of cartilage structure and the subchondral bone due to collagen reaction, extracellular matrix, macromolecules, and proteoglycans usually occurs in TMDs [6, 7]. Internal derangement is a disruption of the internal aspects of TMJ, in which an abnormal relationship exists between the disc and the condyle, fossa, and articular eminence [8].

Advice and medications recommended for TMDs include educating patients on behavior modification and reducing stress, jaw relaxation exercises, soft foods, non-steroidal anti-inflammatory drugs (NSAIDs), splints, and physiotherapy. If surgical intervention is required, management includes arthrocentesis, disc repositioning, or discectomy for TMD patients [1, 9].

This study aimed to compare the results of arthrocentesis with normal saline and arthrocentesis with normal saline and platelet-rich plasma (PRP) by evaluating mouth opening, pain, and bite force as prognostic criteria.

## MATERIAL AND METHODS

**Figure 1:**
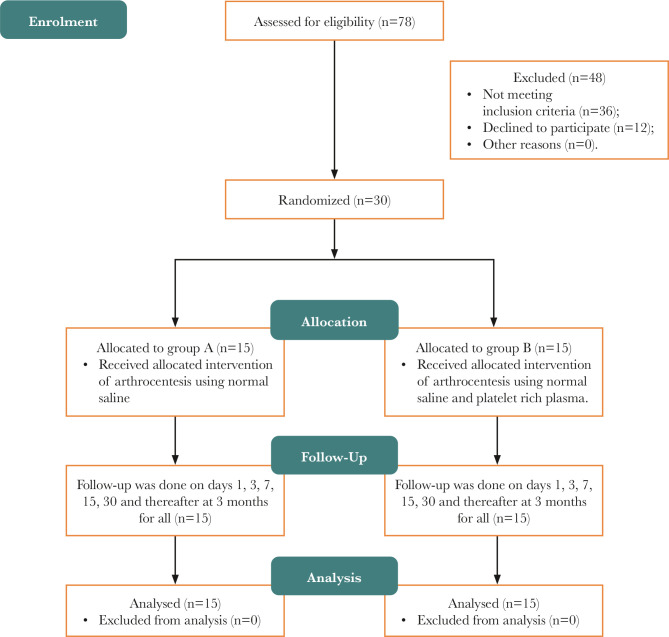
CONSORT Flow Diagram.

This study included 30 patients, with 15 patients randomly selected in group A and 15 patients in group B. All patients were selected from the Outpatient Department (OPD) of Oral and Maxillofacial Surgery, Jamia Millia Islamia, New Delhi, for 24 months (June 2018 to 2020). All patients were selected according to the research and diagnostic criteria (RDC). Arthrocentesis using normal saline was used for Group A, and arthrocentesis using normal saline and PRP was used in Group B (15 patients) (Figure 1).

**Inclusion criteria:** diagnosis by the Research and Diagnostic Criteria and age more than 18 and less than 60.

**Exclusion criteria:** include age less than 18 and above 60, poor overall health of the patient, and diagnosis of connective tissue diseases. Contraindications of using platelet-rich plasma include platelet function disorders, fibrinogen deficiency, and anticoagulation treatment.

### Criteria assessed

- Bite force assessment;
- Pain assessment using Visual Analog Scale (VAS);
- Minimum incisal opening;
- TMJ sounds;
- Operating time;
- Patient compliance.

### Preparation of platelet-rich plasma

5 ml of blood was withdrawn from the patient. Then, the blood was poured into a test tube with sodium citrate anticoagulant (0.5 ml) and centrifuged at an average of 2100 rpm for 15 minutes. After that, the plasma of the first crop was separated using centrifugation at 3500 rpm for 10 minutes to collect the pellet. For each TMJ, 0.8 ml of PRP was injected into 2 ml syringes,

### Operative technique

The temporomandibular region was prepared with an antiseptic solution. The injection point was marked 10 mm forward from the tragus and 2 mm below the trago-canthal line. After blocking the auriculotemporal nerve, the joint was washed with 200 ml of normal saline in group A. In group B, 200 ml of normal saline was injected, and after irrigation with normal saline, platelet-rich plasma was injected. During the procedure, the patient's mouth was wide open. After the injection, the patient was asked to make temporomandibular joint movements slowly.

Postoperatively, the same treatment modality was applied in both groups to all patients after intra-articular injections. Three days after the injection, patients were advised to open their mouths as slowly as possible. Starting with the fourth day onwards, they were asked to open their mouths until reaching the threshold of pain.

Mouth opening was measured using a scale from the incisal edge of the mandibular incisors to the incisal edges of maxillary incisors. Bite force was measured using a bite force measuring device. The bite was measured between upper and lower right maxillary first molars. Three readings were taken, and the mean was calculated for all three readings. Pain measurement was performed using the VAS scale. The observation was made by the same doctor before and after the procedure.

## RESULTS

### Range of opening

The average range of mouth opening (measured between the tip of maxillary and mandibular central incisors) before treatment in groups A and B was 20±1.5 mm. The average interincisal mouth opening before treatment in groups A and B was 16–35 mm. Patients in groups A and B improved mouth opening after 3 months. The average interincisal mouth opening after one month in group A was 25±1.6 mm. After one month, the average incisal opening in group B was 24.2 mm. The average mouth opening after 3 months was 35 in the case of PRP, and in group A it was 30.5. Results pointed out that average mouth opening in group A was similar to mouth opening in group B in the first month, but as time passed, mouth opening in group B improved more than in group A.

### Pain evaluation

Pain evaluation was done in both groups three months after the follow-up. Both groups had a reduction in pain intensity. At the 3-months follow-up, out of 15 patients who underwent PRP therapy, 14 patients had a reduction in pain at their examination. However, of the 15 patients who underwent arthrocentesis with normal saline only, 12 reported a reduction in pain. After treatment, pain relief was significantly more pronounced in group B (treated with PRP). In group A (using normal saline only), after 3 months of follow-up, 8 cases were pain-free, and the other 5 cases had reduced pain intensity. In group B, 9 cases were pain-free, and 5 had reduced pain. Significantly, in the first month after lavage, patients in group B experienced more pain than group A.

**Figure 2:**
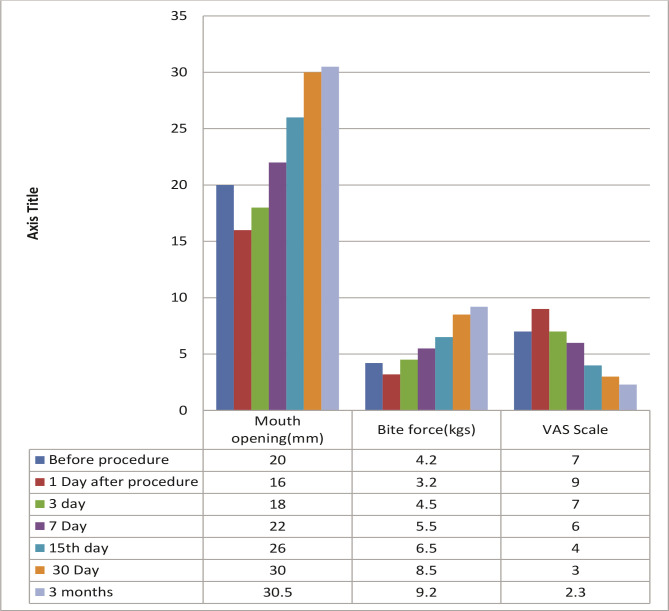
Arthrocentesis using normal saline. Mouth opening, bite force and pain (VAS) measured after arthrocentesis with normal saline. Measurements were taken before the procedure and on 1^st^, 3^rd^, 5^th^, 7^th^, 15^th^, 30^th^ day and 3 months after procedure.

**Figure 3:**
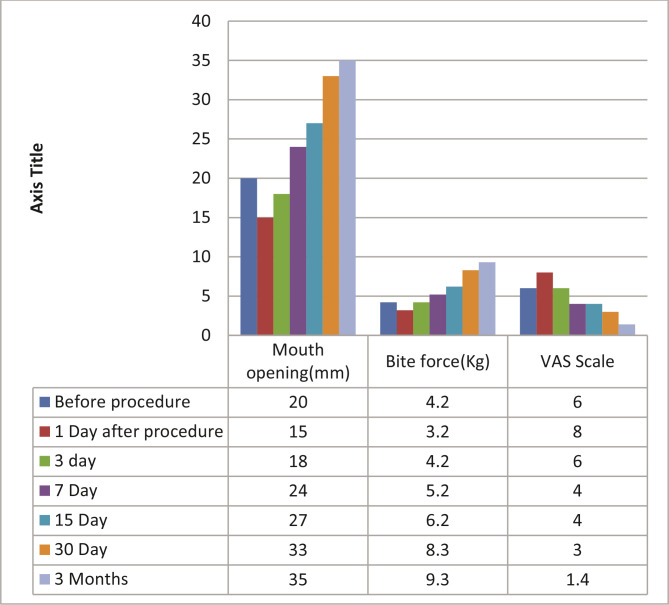
Arthrocentesis using normal saline and platelet rich plasma. Mouth opening, bite force and pain (VAS) measured after arthrocentesis using normal saline and platelet rich plasma (PRP). Measurements were taken before the procedure and on 1^st^, 3^rd^, 5^th^, 7^th^, 15th, 30^th^ day and 3 months after procedure.

**Figure 4:**
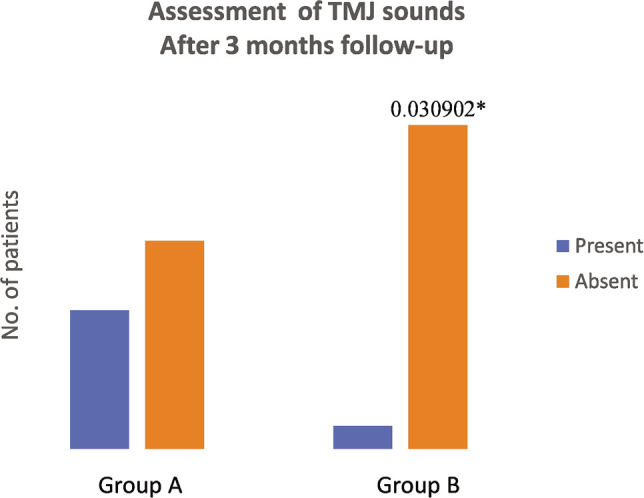
Assessment of TMJ sounds. * – Denotes significant p-value.

According to the VAS scale, both groups experienced pain initially. On the first day after surgery, the pain increased in all patients due to the intervention, but it improved from the second day onwards (Table 1, Figure 2). Also, mouth opening decreased significantly after injection on the first day in both groups (Tables 1 and 2, and Figures 2 and 3), but as time passed, mouth opening improved in both the groups. After 3-months of follow-up, 8 cases out of 15 were pain-free when arthrocentesis with normal saline was done, whereas 10 cases out of 15 were relieved by pain when PRP injections were also given. After three months of follow-up, mouth opening improved in both groups. Improvement in bite force was seen in both groups (Tables 1, 3 and 4), with slightly better results in patients treated with PRP. Improvement in TMJ sounds was noticed in both groups, with PRP being more effective (Figure 4, Table 5).

**Table 1:** Arthrocentesis using normal saline (group A).

		Before procedure	1 day after the procedure	3 days	7 days	15 days	30 days	3 months
**1**	Mouth opening (mm)	20	16	18	22	26	30	30.5
**2**	Bite force (kg)	4.2	3.2	4.5	5.5	6.5	8.5	9.2
**3**	VAS scale	7	9	7	6	5	4	0 in 8 2 in 2 cases. More than 2 in 3 cases and remained the same in 2 cases.
**4**	TMJ sounds (present/absent)	present in 12 cases, absent in 3	-	-	-	-	-	Absent in 7 cases, improved in 4 cases, remained the same in 3 cases

**Table 2:** Arthrocentesis using normal saline and PRP (Group B).

		Before procedure	1 day after the procedure	3 days	7 days	15 days	30 days	3 months
**1**	Mouth opening (mm)	20	15	18	24	27	33	35
**2**	Bite force (kg)	4.2	3.2	3.8	5.2	6.2	8.3	9.34
**3**	VAS scale	6	8	6	5	5	4	0 in 9 cases 1 in 3 cases. More than 2 in 2 cases. It remained same in 1 case.
**4**	TMJ sounds (absent/present)	present in 12 cases, absent in 3	-	-	-	-	-	Absent in 10 cases, improved in 4 cases, remained the same in 1 case

**Table 3:** Mean and Standard Deviation (SD) of the two different treatment modalities.

Treatment modality	Group A		Before procedure	Day 1	Day 3	Day 7	Day 15	Day 30	3 months
**Arthrocentesis by normal saline**	**Mouth opening**	**Mean**	20	16	18	22	26	30	30.5
		**SD**	3.679	2.4511	2.206	1.678	0.496	.425	0.390
	**Bite force**	**Mean**	4.2	3.2	4.5	5.5	6.5	8.5	9.2
		**SD**	0.959	0.827	.814	0.712	.700	.422	.112
	**VAS scale**	**Mean**	7	9	7	6	5	4	2.3
		**SD**	1.934	1.921	1.756	1.323	.897	.316	.892
**Treatment modality**	**Group B**		**Before procedure**	**Day 1**	**Day 3**	**Day7**	**Day 15**	**Day 30**	**3 months**
**Arthrocentesis by normal saline and PRP**	**Mouth opening**	**Mean**	20	15	18	24	27	33	35
		**SD**	2.720	2.4288	1.982	1.345	.7081	0	0
	**Bite force**	**Mean**	4.2	3.2	4.2	5.2	6.2	8.3	9.34
		**SD**	.9431	.7890	.7188	.6117	.5294	0	.234
	**VAS scale**	**Mean**	7	8	6	4	4	3	1.4
		**SD**	1.798	1.656	1.572	1.7471	.2294	0	0

Mean and standard deviation of the two different treatment modalities on the day before the procedure followed by the first day, 3^rd^ day, 7^th^ day, 15^th^ day, 30^th^ day, and 3 months.

**Table 4:** Student's t-test comparing the differences between the two treatment modalities in groups A and B.

Treatment modality	Group A		Before procedure	Day 1	Day 3	Day 7	Day 15	Day 30	3 months
**Group A vs. Group B**	**Mouth opening**	**p-value**	1.0000	.2712	1.000	.0012*	.0001*	.0001*	.0001*
	**Bite force**	**p-value**	1.0000	1.0000	.2938	.2261	.1963	.0771	.0458*
	**VAS scale**	**p-value**	1.0000	0.1380	.1115*	.0014*	.0003*	.0001*	.0005*

* – Denotes significant p-value.

**Table 5:** TMJ sounds.

Before treatment			
**TMJ sounds**	**Group A**	**Group B**	**P-value**
**Present**	12	12	1
***After 3 months***			
**Absent**	3	3	
**TMJ sounds**	**Group A**	**Group B**	**p-value**
**Present**	6	1	.030902*
**Absent**	9	14	

* – Denotes significant p-value. The Chi-square test showed significant values for the absence of TMJ sounds after 3 months.

The time consumed in the first group was significantly less than in the second group, as the second group required withdrawal of blood and PRP preparation, but compliance in group A was slightly better.

## DISCUSSION

Comparing PRP combined with arthrocentesis to arthrocentesis alone elicited more promising and coherent results. Reports by Kilic et al. revealed superior masticatory efficiency in patients treated with PRP injections, and cone-beam computed tomography (CBCT) evaluation also validated two-fold improved reparative remodeling in osteoarthritis patients [5]. However, a study by Lin et al. elicited similar reparative remodeling in both treatments (PRP injections and PRP arthrocentesis) [6].

According to Mustafa et al. [7], there was a higher efficacy of intra-articular PRP injections than arthrocentesis for the treatment of reducible disc displacement of TMJ [10]. Recalcitrant TMD cases are better managed by intraarticular PRP injection and arthrocentesis, resulting in symptoms diminution and functional amelioration [8]. Another study also highlighted the significance of intra-articular PRP injections in TMD management [11, 12]. The presence of masticatory muscle pain and/or TMJ inflammation can play a role in maximum bite force [13]. However, the mechanisms involved in this process are not well understood. According to Khan et al. [14], maximum bite force was higher in young males than in young females. Also, bite force tends to decrease as age progresses.

## CONCLUSION

According to the VAS scale, all patients experienced pain in both groups initially. On the first day after surgery, the pain increased and mouth opening reduced because of the intervention, but it improved from the second day (Table 3, Figures 1 and 2). After three months, 8 cases out of 15 were pain-free with arthrocentesis with normal saline, whereas 10 cases out of 15 were relieved by pain with PRP injections (Tables 2, 3 and 4). Mouth opening improved in both groups. Improvement in bite force was seen in both groups, with a slight improvement in patients treated by PRP. Improvement in TMJ sounds was noticed in both groups, with PRP being more effective (Table 5, Figure 3). Significantly, the time consumed in the first group was less than the second group, as the second group required blood withdrawal and PRP preparation, but compliance in group A was slightly better.
